# L-proline mixed with sodium benzoate as sustainable inhibitor for mild steel corrosion in 1M HCl: An experimental and theoretical approach

**DOI:** 10.1038/s41598-018-24143-2

**Published:** 2018-05-10

**Authors:** Mosarrat Parveen, Mohammad Mobin, Saman Zehra, Ruby Aslam

**Affiliations:** Corrosion Research Laboratory, Department of Applied Chemistry, Faculty of Engineering and Technology, Aligarh Muslim University, Aligarh, 202 002 India

## Abstract

Following standard experimental (gravimetric measurements, potentiodynamic polarization measurements, electrochemical impedance measurements, spectroscopic measurements, scanning electron microscopy technique) and theoretical (DFT) approach, inhibition effect of L-proline (LPr) and LPr mixed with sodium benzoate (LPr + NaBenz) for mild steel (MS) corrosion in 1M HCl was studied at 30, 40, 50 and 60 °C. The concentration of LPr was varied between 100–600 ppm, whereas that of NaBenz was fixed at 10 ppm. LPr lowered the corrosion rates of MS to a considerable extent. Corrosion mitigating efficacy of LPr is synergistically enhanced on adding NaBenz at all concentrations. Evaluation of polarization parameters suggested that both LPr and LPr + NaBenz act as mixed type inhibitor with more control on cathodic reaction whereas impedance parameters suggested inhibition of metal corrosion by adsorption at the MS/solution interface. Surface microscopic examination of corroded and uncorroded MS coupons supported the protective effect of adsorbed inhibitor layer at the MS surface. Spectroscopic studies are suggestive of the complex formation between inhibitor molecules and the metal. When LPr is combined with NaBenz, the corrosion inhibition rate was improved greatly. Corrosion mitigating efficacy of LPr or LPr mixed with NaBenz obtained by different techniques are in good agreement and correlate well with theoretical quantum chemical descriptors.

## Introduction

As an important metal in the world, mild steel (MS) is the material of choice in petroleum and machinery industries due to its perfect mechanical workability and economic feasibility^1,2^. In many industrial applications, acid cleaning treatment of the MS components is often carried out to remove rust and scales formed after several working cycles^3^. During acid cleaning treatment MS surface may be severely corroded if used without appropriate corrosion inhibitor. The use of many heterocyclic organic compounds having multiple bonds, aromatic rings or hetero atoms like nitrogen, oxygen, sulfur is one of the practically acknowledged practices for corrosion protection of MS in acidic media, as well as for reduction of acid consumption occurring during the process since long time^4–9^. These inhibitors control metal dissolution by adsorbing on the corroding sites of steel and prevent its exposure to the corrosive environment^10^. Inhibitor adsorption process depends on structure of inhibitor and nature of corrosive environment^11^. However, majority of the heterocyclic organic compounds though yielded high inhibition efficacy, their applications is limited by: (i) high cost of production, (ii) toxic by-products often formed during their production leading to environmental concern and (iii) the specificity of action associated with the use of single compound inhibitors. Therefore, corrosion inhibitors should not only be efficient but cost effective and environmentally benign^12,13^. Amino acids containing π electrons and heteroatoms in their molecules satisfy these criteria and thus proving as a potential source of new corrosion inhibitors^14^. However, their application is hindered due to their moderate to low inhibition efficacy. Enhancing the performance of individual inhibitor by adding certain additives has turned out to be an effective method to improve the inhibition performance or to decrease the required dosage of the inhibitor and to expand the application of the inhibitor in acidic media^15,16^.

Following the same line, and equipped with the encouraging results on our previous studies on amino acids and surfactant additives^8,17,18^ as MS corrosion inhibitor in acid medium, the focus of the present study is to gain some insight into the effect of LPr and LPr mixed with NaBenz as safe inhibiting formulation for MS in 1M HCl solution.

## Experimental

### Metal specimen and test solution

**Figure 1:**
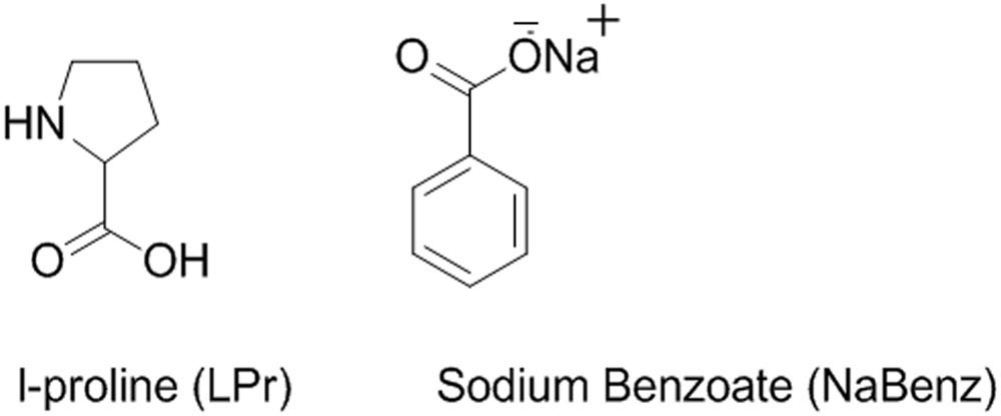
Molecular structure of studied inhibitors.

Chemical composition of MS, as analysed by optical emission spectrometer, (in weight%) chosen in the study: C −0.20, S −0.11, P −0.098, Mn −0.53, Si −0.036 and the remaining Fe. Dimension of working electrode for electrochemical experiments is 1.0 cm^2^ whereas, rectangular specimens of dimension 2.5 × 2.0 × 0.03 cm with exposed surface area of 10.27 cm^2^ were used during gravimetric studies. Specimens were abraded using emery papers (from grade 320–1200), degreased with 1:1 ethanol/water mixture, rinsed with distilled water and finally dried in warm air and used with no further storage. L-proline (Pyrrolidine-2-carboxylic acid), molecular mass 115.13 g mole^−1^], and NaBenz were used as received. Experiments were done in 1M HCl solutions (aerated and unstirred) without and with different concentrations of LPr (100–600 ppm) and fixed concentration (10 ppm) of NaBenz. Molecular structure of LPr and NaBenz is given in Fig. 1.

### Electrochemical measurements

**Figure 2:**
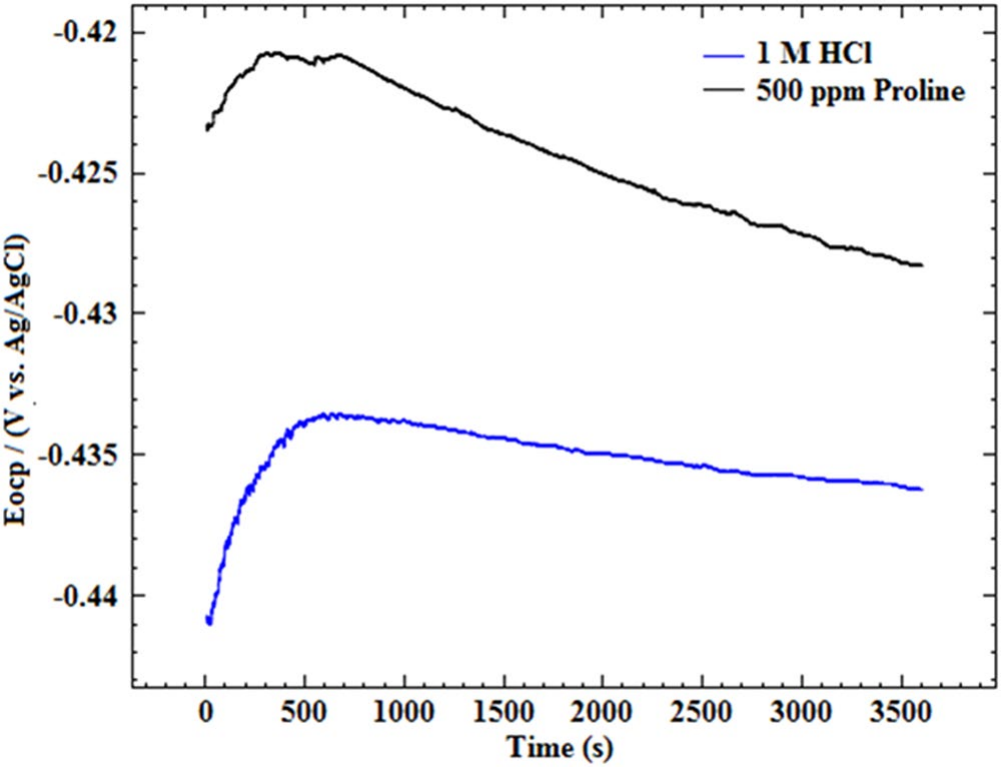
Variation of *E*_OCP_-time curves for MS in uninhibited and inhibited 1M HCl solution.

Electrochemical measurements were performed on AutolabPotentiostat/Galvanostat, model 128 N with inbuilt impedance analyser FRA2 module. The electrochemical experiment consisted of a three electrode configuration. Mild steel, platinum foil and Ag/AgCl were used as working, counter and reference electrodes, respectively. The working electrode, which was a circular mild steel specimen with exposed surface area of 1.0 cm^2^, was prepared following the same procedure as mentioned for gravimetric experiments. All the measurements were conducted at the end of 30 min immersion at 30 ± 2 °C using a temperature-controlled water bath to obtain steady-state open circuit potential (OCP). Steady state potential was confirmed when no significant change in rest potential was detected (Fig. 2).

Electrochemical impedance spectroscopy (EIS) experiments were performed at OCP within frequency range 10^5^–10^−2^ Hz with signal amplitude perturbation of 10 mV. Charge transfer resistance (*R*_ct_) values were used to calculate% inhibition efficiency, following the relationship:

1 $$({\eta }_{{\rm{Rct}}} \% )={\frac{{Rct}-Rct}{Rct}}^{o}\times {\rm{100}}$$

where, *R*_ct_ and *R*_ct_° are the charge transfer resistance with and without LPr or LPr + NaBenz, respectively. Potentiodynamic polarization (PDP) studies were carried out in the potential range ±250 mV versus corrosion potential. The scan rate was 0.001 Vs^−1^. In order to confirm the reproducibility of the systems electrochemical experiments were also performed in triplicate. The measured *i*_corr_ values were used to calculate inhibition efficiency using the following equation:

2 $$ \% {\eta }_{{\rm{i}}}=\frac{{{\rm{i}}}_{{\rm{corr}}}^{^\circ }-{{\rm{i}}}_{{\rm{corr}}}}{{{\rm{i}}}_{{\rm{corr}}}^{^\circ }}\times {\rm{100}}$$

where, *i*°_corr_ and *i*_corr_ are the corrosion current density in the absence and presence of inhibitor.

### Gravimetric measurements

MS Specimen (dimension: 2 × 2.5 × 0.03 cm) were considered for gravimetric experiments. These were prepared, cleaned and immersed in test solutions at temperatures 30–60 °C following procedures mentioned in our earlier publications^8,19,20^. After desired period of immersion the specimens were moved out, the corrosion products were removed mechanically^21^ by scrubbing gently with bristle brush, washed, dried and final weight was recorded. The weight loss was taken to be the difference between the weight of the coupons at a given time and its initial weight. Tests were performed in triplicate and the data showed good reproducibility. Corrosion rate (*ѵ*, mg cm^−2^ h^−1^), inhibition efficiency (*η*_w,_%) and surface coverage (*θ*) was obtained following the relationships:

3 $$\nu =\frac{{w}_{1}-{w}_{{2}}}{At}$$

4 $$\theta =\frac{{\nu }_{{o}}-{\nu }_{{i}}}{{\nu }_{{o}}}$$

5 $${\eta }_{{\rm{w}}}\,( \% )=\frac{{\nu }_{{o}}-{\nu }_{{i}}}{{\nu }_{{o}}}\times 100$$

where *w*_1_ and *w*_2_ - the weight of the MS coupons before and after immersion in the test solution, *A* - area of coupon, *t*- immersion time, and *ν*_o_ and *ν*_i_- the corrosion rates in absence and presence of inhibitor.

To judge that effect of NaBenz on corrosion inhibition behavior of LPr is synergistic in nature, synergism parameter, *S*_θ_ was computed following the relationship^22^:

6 $${{\rm{S}}}_{{\rm{\theta }}}=1-{{\rm{\theta }}}_{1+2}/1-{{\rm{\theta }}^{\prime} }_{1+2}$$

7 $${{\rm{\theta }}}_{1+2}=({{\rm{\theta }}}_{1}+{{\rm{\theta }}}_{2})-({{\rm{\theta }}}_{1}{{\rm{\theta }}}_{2})$$

where, *θ*_1_ and *θ*_2_ - surface coverage by LPr and NaBenz, respectively and *θ*′_1+2_- measured surface coverage by both the LPr and NaBenz. In general, *S*_θ_ > 1 suggests a synergistic effect whereas *S*_θ_ < 1 implies that antagonistic behaviour prevails, which may lead to competitive adsorption.

### UV-Visible spectroscopy study

UV-visible absorption spectra were obtained for 1M HCl solution containing optimum concentration of LPr + NaBenz prior to and after the MS immersion for 6 h at 30 °C. Then, the coupons were removed and rinsed with distilled water, dried in air and finally analyzed using Perkin Elmer spectrophotometer, Lambda 25.

### FT-IR spectroscopy study

FT-IR spectroscopy was used to identify the functional groups in LPr and NaBenz. FT-IR Spectra were recorded using spectrometer from Perkin Elmer (‘Spectrum Two’ with spectral resolution 0.5 cm^−1^) equipped with Spectrum Software in the frequency range 4000 to 400 cm^−1^ following KBr disc technique. The other sample, the film of LPr + NaBenz formed on the steel surface, was obtained by immersing the steel in 1M HCl solution containing 500 ppm + 10 ppm NaBenz for 6 h immersion.

### Surface analysis of specimen

To record surface morphology of uncorroded and corroded (in uninhibited and inhibited acid) MS specimen at 30 °C SEM (Model: JEOL JSM-6510LV) was used. For SEM studies MS surface was prepared by immersing in 1M HCl solution in absence and presence of 500 ppm of LPr and LPr (500 ppm) + NaBenz (10 ppm) at 30 °C. After 6 *h* of immersion the specimens were retrieved, thoroughly washed with distilled water, dried in air and examined under SEM.

### Density functional theory

In order to understand the inhibitory actionof the investigated compund a number of quantum chemicaldescriptors were computed by Density Functional Theory (DFT) using ORCA programme (version 3.0.3) involving Becke’s three parameter hybrid functional and Lee-Yang-Paar correlation functional (B3LYP) combined with def2-SVP basis set to obtain the full optimized geometry^23–25^. The calculated descriptors are the energy of highest occupied molecular orbital (*E*_HOMO_), the energy of lowest unoccupied molecular orbital (*E*_LUMO_), the separation energy (Δ*E*_LUMO-HOMO_), total energy (*E*_t_) absolute electronegativity (*χ*), absolute hardness (*η*), number of electron transferred (Δ*N*), the Mulliken charges on the atoms and molecular electrostatic potential maps (MEP) in the range from deepest red colour to deepest blue colour to help in the explanation of the experimental data obtained for the corrosion process.

## Results and Discussion

### Potentiodynamic polarization measurements

**Figure 3:**
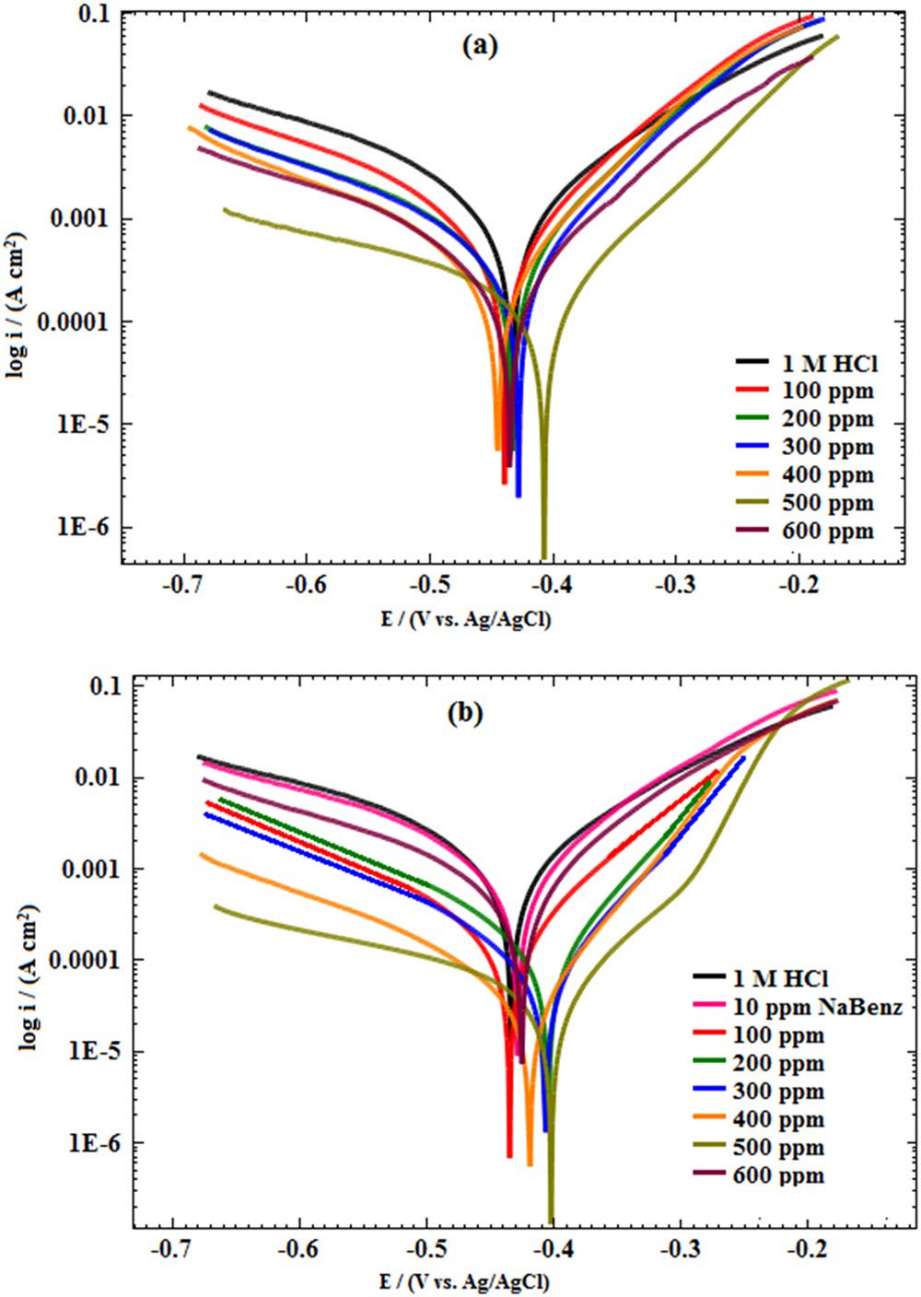
Potentiodynamic polarization curves of MS in 1M HCl solution without and with different concentrations of LPr and LPr + 10 ppm NaBenz.

Potentiodynamic polarization experiments were undertaken to distinguish the effect of different concentration of LPr or LPr mixed with NaBenz on the anodic and cathodic corrosion reactions. The polarization curves for the MS sample in 1M HCl without and with LPr and LPr + NaBenz are displayed in Fig. 3a,b. Polarization parameters including the values of anodic Tafel slope (*β*_a_), cathodic Tafel slope (*β*_c_), corrosion potential (*E*_corr_) and corrosion current density (*i*_corr_) are listed in Table 1. It is suggested that if the displacement in *E*_corr_ is >85 mV with respect to the *E*_corr_ of blank solution, the chemical inhibitor can be seen as an anodic or cathodic type inhibitor, and if the displacement in *E*_corr_ is <85 mV, the inhibitor can be seen as a mixed type^26,27^. From the data given in Table 1, it is clear that the magnitude of *E*_corr_ < 85 mV implying that both LPr and LPr + NaBenz act as mixed type corrosion inhibitor. In other words, LPr and LPr + NaBenz could reduce anodic dissolution of MS and suppress the cathodic hydrogen evolution reaction. The results in Table 1 showed that the cathodic slopes of LPr and LPr + NaBenz decreased slightly more than the anodic slopes, which indicated that the decrease in cathodic reaction rates is more obvious than the corresponding anodic ones. This observation also indicated that LPr and LPr + NaBenz are a mixed type inhibitor with slightly more control of cathodic reaction. From Table 1, it is found that compared to the blank solution (1M HCl), the *i*_corr_ values reduces appreciably by addition of LPr or LPr + NaBenz. This observation suggests that the rate of MS dissolution was retarded by the formation of a protective inhibitor film on the metal surface which created a barrier between the steel surface and the aggressive medium^28^. Moreover, the value of *i*_corr_ for the mixture of LPr + NaBenz is around 0.045 × 10^−3^ A cm^−2^, much lower than that for LPr (i.e. 0.288 × 10^−3^ A cm^−2^) at 500 ppm. The inhibition efficiency (*η*_PDP_%) of the LPr + NaBenz is up to 90.7%, higher than that of LPr (i.e. 83.9%) alone. These results suggest that the inhibitive performance of the LPr + NaBenz is better than that of LPr or NaBenz alone. Inhibition efficiency obtained by potentiodynamic polarization measurements are consistent with the one obtained by EIS.

**Table 1:** PDP Parameters for MS in 1M HCl in the absence and presence of different concentrations of LPr and LPr + 10 ppm NaBenz at 30 °C.

*C* (ppm)	*E*_corr_ (V vs. Ag/AgCl)	*β*_a_/(V dec^−1^)	*β*_c_/(V dec^−1^)	*i*_corr_ × 10^−3^(A cm^−2^)	*η*_PDP_ (%)
1M HCl	−0.43	0.162	0.130	1.20	—
LPr					
100	−0.440	0.111	0.084	0.436	63.7
200	−0.433	0.122	0.074	0.325	72.9
300	−0.431	0.118	0.076	0.277	76.9
400	−0.445	0.118	0.079	0.233	80.6
500	−0.412	0.288	0.102	0.192	83.9
600	−0.435	0.131	0.089	0.218	81.8
LPr + 10 ppm NaBenz					
10 ppm NaBenz	−0.433	0.143	0.109	1.141	5.0
100	−0.436	0.106	0.070	0.130	73.0
200	−0.405	0.107	0.069	0.107	77.8
300	−0.408	0.109	0.069	0.07	85.7
400	−0.405	0.242	0.077	0.06	87.6
500	−0.403	0.196	0.089	0.045	90.7
600	−0.414	0.119	0.067	0.092	81.1

### Electrochemical impedance spectroscopy

**Figure 4:**
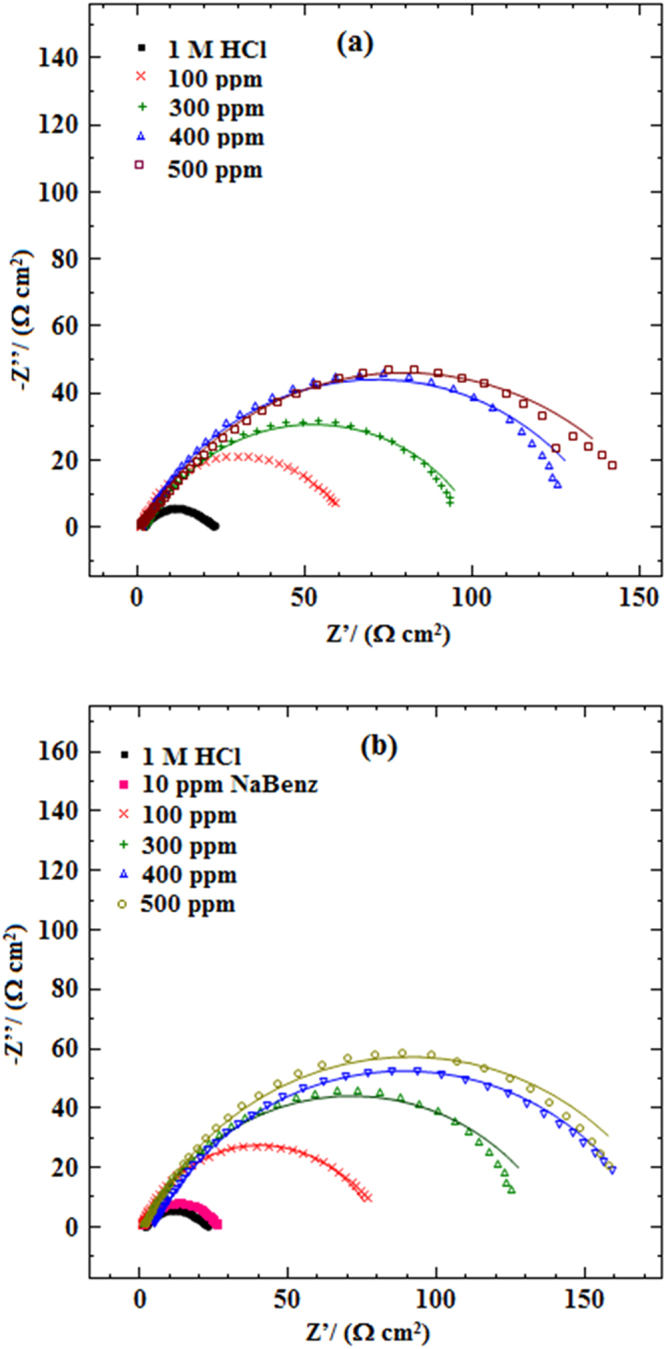
Nyquist plots of MS in 1M HCl solution without and with different concentrations of (**a**) LPr and (**b**) LPr + 10 ppm NaBenz.

**Figure 5:**
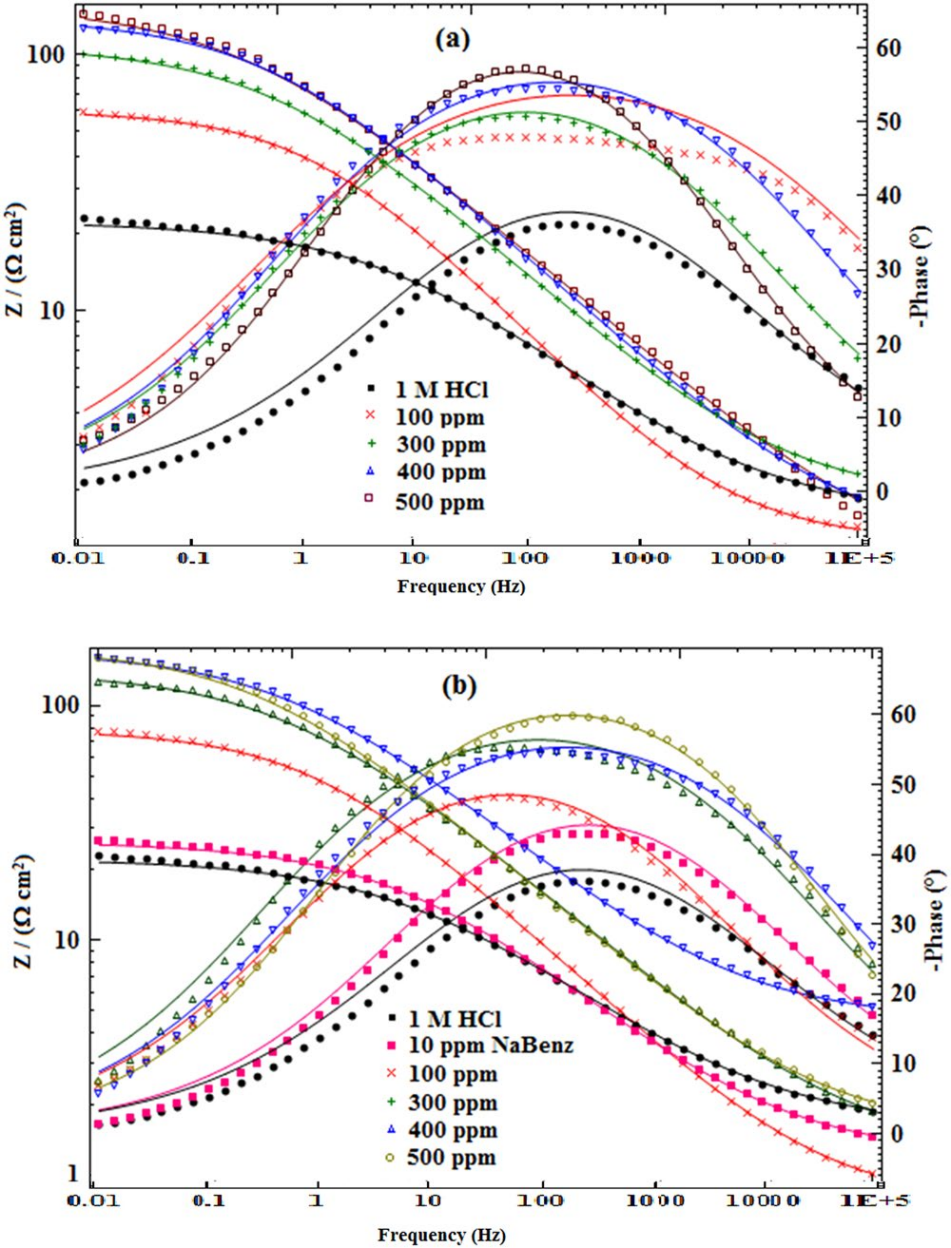
Bode plots of MS in 1M HCl solution without and with different concentrations of inhibitors (**a**) LPr and (**b**) LPr + 10 ppm NaBenz.

Corrosion inhibition effect of LPr or LPr/NaBenz concentration on the impedance behavior of MS in test solution (1M HCl) has been evaluated. Nyquist and Bode plots related to MS immersed in uninhibited HCl solution and acid solution with different concentrations of LPr or LPr + NaBenz are shown in Figs 4, 5. Table 2 lists the corresponding impedance parameters. The depressed semicircle of Nyquist plots for 1M HCl without and with inhibitor (Fig. 4) can be correlated to the roughness and inhomogeneity of surface created due to the adsorption of the inhibitor molecules on metal surface^29,30^. It is observed from curves that the diameter of the semicircles increased with increasing LPr or LPr + NaBenz concentration, this occurrence specifies that corrosion is a charge transfer process mainly^31^. The notable point is that in presence of inhibitor, the Nyquist plots are similar and this means that protection mechanism does not change during the whole process^32^.

**Table 2:** EIS parameters of MS in 1M HCl in absence and presence of varying concentrations of LPr and LPr + 10 ppm NaBenz inhibitors at 30 °C.

*C* (ppm)	*R*_s_ (Ω cm^2^)	*R*_ct_ (Ω cm^2^)	*χ*^2^ × 10^−2^	*CPE*		*C*_dl_ × 10^−3^ (Fcm^−2^)	*η*_EIS_ (%)
				*Y*_0_ × 10^−3^ (Ω^−1^s^n^cm^−2^)	*n*		
1M HCl	1.473	21.22	2.3	0.16	0.9935	0.155	—
LPr							
100	1.21	60.428	2.5	0.159	0.9961	0.156	64.9
200	0.61	81.202	1.5	0.151	0.9956	0.148	73.8
300	3.05	96.79	2.7	0.106	0.9953	0.104	78.1
400	1.48	133.26	5.7	0.098	0.9958	0.962	84.1
500	17.11	156.29	2.1	0.091	0.9961	0.891	86.4
600	14.13	117.66	1.8	0.081	0.9963	0.080	81.9
LPr + 10 ppm NaBenz							
10 ppm NaBenz	1.18	21.9	2.1	0.155	0.9949	0.152	3.1
100	0.61	81.20	2.2	0.153	0.9956	0.15	73.9
200	3.05	96.79	3.1	0.106	0.9953	0.104	78.1
300	1.48	133.26	1.6	0.108	0.9958	0.106	84.1
400	17.10	156.29	2.9	0.118	0.9961	0.117	86.4
500	2.59	169.51	2.7	0.116	0.9959	0.114	87.5
600	0.60	138.64	3.4	0.120	0.9940	0.117	84.7

**Figure 6:**
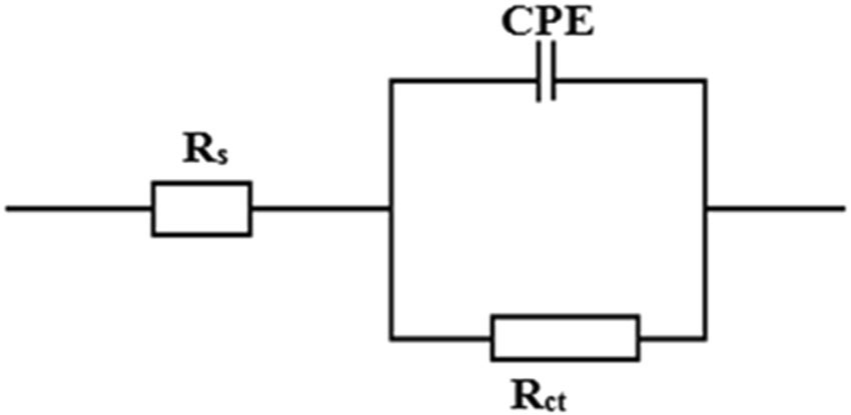
Equivalent circuit model used to fit the impedance measurement data for MS in 1M HCl (*R*_s_ = solution resistance, *R*_ct_ = charge-transfer resistance, and *CPE* = constant phase element).

The equivalent circuit^33,34^ which was used to analyze the impedance data is shown in Fig. 6. In referred equivalent circuit, *R*_s_ is the solution resistance, *R*_ct_ is the charge transfer resistance, and CPE is the constant phase element of the surface layer. A good fit with this model was observed with our experimental data (Fig. 4). It is observed that the fitted data match with the experimental data, with the chi-square value (*χ*^2^). In the fitted Nyquist and Bode plots, Figs 4, 5, the symbols represent the experimental data, whereas solid lines show the best fits. To get more accurate semicircle fit CPE is substituted for double layer capacitance, *C*_dl_ as metal/solution interface does not correspond to an ideal capacitor. Impedance of a CPE is given by the expression^31^:

8 $${{\rm{Z}}}_{CPE}=\frac{1}{{Y}_{o}{(j\omega )}^{n}}$$

where *Y*_o_ is the magnitude of CPE, *j* is the imaginary number and is equal to the square root of −1, *ω* is the angular frequency in Rads^−1^ (*ω* = 2*πf*_max_) and *n* corresponds to the phase shift, which is related to the inhomogeneties of the double layer. For *n* = 0, *Z*_CPE_ represents a resistance with R = *Q*^−1^, for *n* = 1 a capacitance with *C* = *Q* and for *n* = −1 an inductance with *L* = *Q*^−1^. The *C*_dl_ for a circuit including CPE can be calculated using the equation (9)^35^:

9 $${{\rm{C}}}_{{\rm{dl}}}={Y}_{o}{({\omega }_{\max })}^{n-1}$$

where, *ω*_max_ = 2*πf*_max_ (*f*_max_ denotes maximum frequency at which the imaginary component of the impedance has a maximum).

The main parameters obtained from EIS measurements are *R*_ct_ and *C*_dl_. A decrease in *C*_dl_ values with accompanying increase in *R*_ct_ values for inhibitor concentration up to 500 ppm suggest that corrosion inhibition performance of LPr or LPr mixed with NaBenz for MS in 1M HCl solution is due to the increased surface coverage as well as increased thickness of adsorbed layer. The increase in LPr concentration above 500 ppm does not have a positive inhibitive effect.

The increased inhibition effect of LPr with increasing concentration or by the addition of NaBenz, as visualized by Nyquist plots, is further confirmed by the corresponding Bode diagrams (Fig. 5). In Bode modulus diagram, for whole frequency range, the impedance modulus is observed to increase with increasing LPr concentration or addition of NaBenz, whereas in Bode phase angle diagram a more negative value of phase angle at high frequency is noticed with increasing LPr concentration or addition of NaBenz. An increase in the values of absolute impedance at low frequencies with increase in LPr concentrations or addition of NaBenz to the LPr indicates lower corrosion rates or higher protection of MS in inhibited acid solution. Further, more negative values of phase angle at high frequencies with increase in LPr concentrations or addition of NaBenz to the LPr indicates superior inhibitive behaviour at higher concentration of LPr or for LPr mixed with NaBenz.

### Weight loss measurements

#### Effect of concentration and temperature

**Figure 7:**
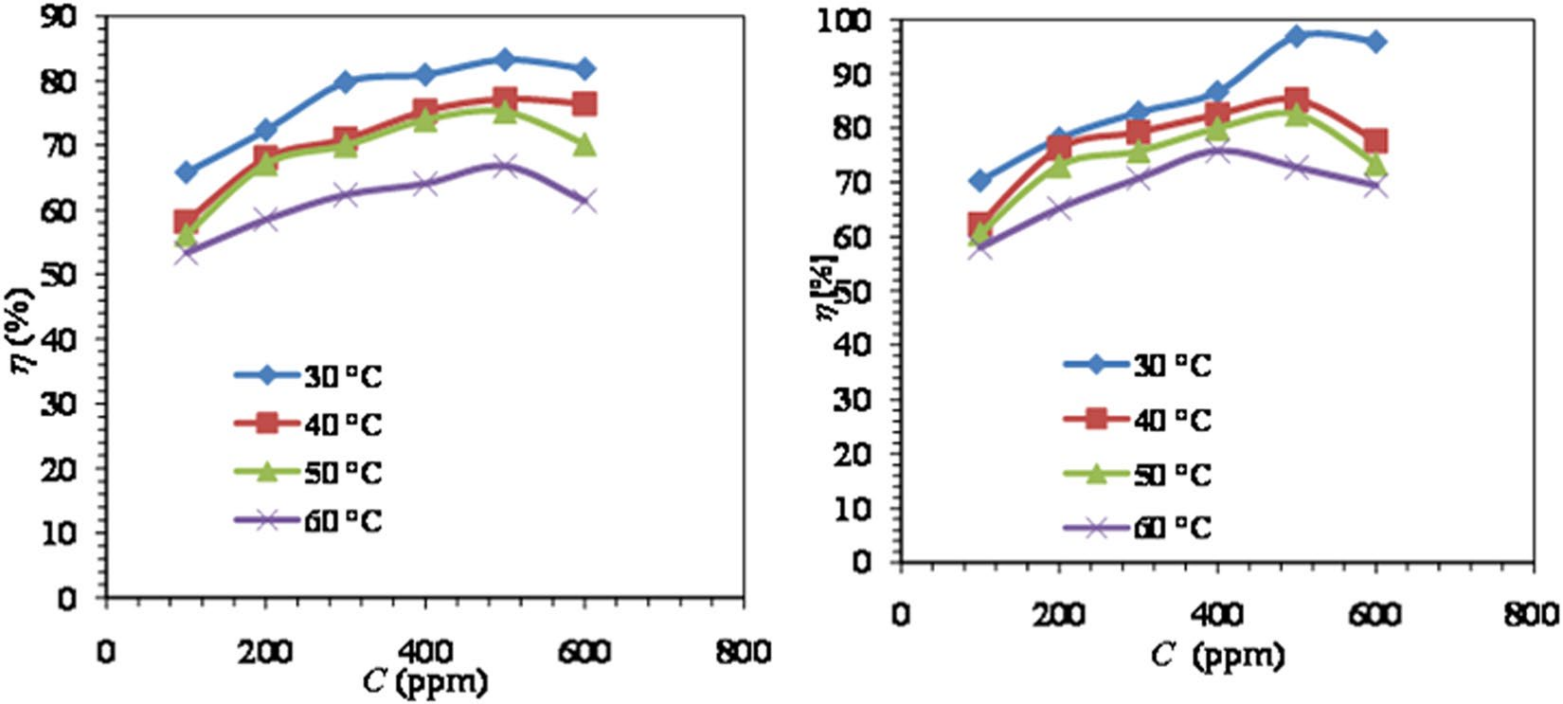
Variation of inhibition efficiency (*η*_w_%) vs. temperature (°C) (**a**) LPr and (**b**) LPr + 10 ppm NaBenz.

The corrosion parameters of MS in 1M HCl without and with varying concentrations of LPr or LPr + 10 ppm NaBenz was evaluated using weight loss technique. To observe the effect of temperature the weight loss studies were performed at temperatures 30, 40, 50 and 60 °C and the results are listed in Table 3. Data in Table 3 suggest the inhibitor LPr to be acting as moderate corrosion inhibitor for MS corrosion in 1M HCl solution, the inhibition efficiency of inhibitor being both concentration and temperature dependent. The increase in LPr concentrations causes an increase in inhibition efficiency till it reaches a maximum value of 82.5% at LPr concentration of 500 ppm at 30 °C, a further increase in LPr concentration resulted in a little lowering of the inhibition efficiency (81.8%) due to desorption of some of the LPr molecules (Fig. 7a,b). The inhibition efficiency of LPr alone in combination with organic salt (NaBenz) over the range of concentrations studied is higher than that reported for some amino acids and amino acids + additives, in the literature^14,36–42^ (see Table 4). Compared to previously studied amino acids based corrosion inhibitors; the present compound yields much higher inhibition efficiency at very low concentration. This aspect adds to the practicality of using the present system (LPr + NaBenz) under investigation. However, an increase in temperature resulted in the lowering of inhibition efficiency at all the studied concentrations, suggesting physical adsorption. In such type of adsorption the existing weak Vander Waal’s forces tend to diminish at elevated temperatures^43^.

**Table 3:** Corrosion parameters for MS in 1M HCl solution in the absence and presence of different concentrations of LPr and LPr + 10 ppm NaBenz at 30–60 °C temperatures obtained from weight loss measurements.

*C* (ppm)	30 °C		40 °C		50 °C		60 °C		30 °C	40 °C	50 °C	60 °C
	*υ* (mg cm^−2^ h^−1^)	*η* (%)	*υ* (mg cm^−2^ h^−1^)	*η* (%)	*υ* (mg cm^−2^ h^−1^)	*η* (%)	*υ* (mg cm^−2^ h^−1^)	*η* (%)	*S*_θ_			
LPr												
0	1.11 ± 0.05		3.9 ± 0.40		5.16 ± 0.40		12.73 ± 0.75					
100	0.38 ± 0.02	65.8	1.6 ± 0.09	58.2	2.26 ± 0.04	56.2	5.94 ± 0.40	53.3	—	—	—	—
200	0.31 ± 0.02	72.4	1.3 ± 0.06	68.0	1.69 ± 0.04	67.2	5.28 ± 0.20	58.5	—	—	—	—
300	0.22 ± 0.01	79.8	1.1 ± 0.03	71.0	1.55 ± 0.07	70.0	4.79 ± 0.14	62.3	—	—	—	—
400	0.21 ± 0.01	80.9	0.97 ± 0.03	75.3	1.34 ± 0.03	74.0	4.57 ± 0.10	64.0	—	—	—	—
500	0.18 ± 0.01	82.5	0.89 ± 0.03	77.3	1.28 ± 0.10	75.2	4.23 ± 0.21	66.7	—	—	—	—
600	0.20 ± 0.01	81.8	0.93 ± 0.02	76.4	1.53 ± 0.07	70.2	5.01 ± 0.24	61.4	—	—	—	—
LPr + 10 ppm NaBenz												
10 ppmNaBenz	1.07 ± 0.06	3.5	3.71 ± 0.32	5.7	4.7 ± 0.21	7.9	11.46 ± 0.59	9.9				
100	0.329 ± 0.02	70.3	1.5 ± 0.02	62.3	2.0 ± 0.09	60.6	5.34 ± 0.15	58.0	1.11	1.05	1.02	1.00
200	0.243 ± 0.01	78.1	0.93 ± 0.08	76.3	1.4 ± 0.09	73.1	4.42 ± 0.34	65.2	1.21	1.27	1.13	1.07
300	0.189 ± 0.01	82.9	0.81 ± 0.04	79.2	1.3 ± 0.09	75.7	3.72 ± 0.30	70.7	1.14	1.32	1.14	1.16
400	0.147 ± 0.01	86.7	0.68 ± 0.03	82.6	1.0 ± 0.02	79.9	3.07 ± 0.24	75.8	1.38	1.33	1.19	1.34
500	0.034 ± 0.002	96.9	0.58 ± 0.02	85.2	0.9 ± 0.02	82.5	3.46 ± 0.30	72.7	5.25	1.45	1.31	1.10
600	0.045 ± 0.002	95.9	0.88 ± 0.03	77.6	1.4 ± 0.07	73.4	3.88 ± 0.30	69.4	4.28	0.99	1.03	1.13

**Table 4:** Comparison of the inhibition efficiency of studied inhibitor with other amino acids obtained for MS in acidic media reported in the literature.

S. No.	Compounds	*C* (ppm)	*T* (°C)	*η* (%)	ref.
1.	l-proline	500	30	82	present study
2.	alanine	8909	30	80	^14^
3.	glycine	7507	30	78	^14^
4.	leucine	13107	30	91	^14^
5.	Methionine	1492	30	71	^36^
6.	Alanine	447	30	75	^37^
7.	Valanine	117.48	25	60	^38^
8.	L-lysine	1468	30	70	^39^
9.	Tryptophan	2042	25	91	^40^
10.	Tryptophan	8168.8	25	94	^41^
11.	L-Histidine	500	30	71	^42^
12	l-proline + 10 ppm NaBenz	500	30	96	present study
13	Methionine + 746 ppm KI	746	30	98	^36^

The corrosion inhibition behaviour of LPr at temperatures 30–60 °C was also examined in presence of 10 ppm each of NaBenz and the results are presented in Table 3. The presence of 10 ppm of NaBenz further enhanced the inhibition efficiency of LPr at all the studied concentration and temperature. It is also obvious that the NaBenz was more effective in 1M HCl at all concentrations and attained maximum efficiency more rapidly. The maximum inhibition efficiency of 82.5% exhibited by LPr at 500 ppm at 30 °C was increased to 96.9% in presence of NaBenz. The observed increase in inhibition efficiency is more than either of LPr or NaBenz alone indicating a synergistic effect between LPr and NaBenz. Values of synergism parameter (S_θ_) calculated for LPr + NaBenz at all temperatures are greater than unity (Table 3) indicating that the increased inhibition efficiency of LPr resulting from the addition of NaBenz is synergistic in nature and proves that addition of a very small concentration of NaBenz can significantly improve the adsorption of LPr on the MS surface. Also when the inhibition efficiency (*η*_EIS_) decreases with increase in temperature as observed in this study (Table 3), it points straight to physical adsorption mechanism^44^.

#### Statistical Analysis

Statistical analysis was employed through ANOVA at a confidence level of 95% i.e. a significance level of α = 0.05 and the ANOVA results are given in Table 5. From this table, it is clear that the p-values are <0.05. From p-values, it is concluded that there is significant difference in inhibition efficiencies obtained by increasing concentration.

**Table 5:** ANOVA for inhibition efficiency of l-Proline in 1M HCl (at 95% confidence level).

Source of Variation	***SS***	***df***	***MS***	***F***	***P-value***
Inhibitor concentration	1184.01	4	296.00	324.91	2.26E-14
Total	1184.01	4			

#### Effect of immersion time

**Figure 8:**
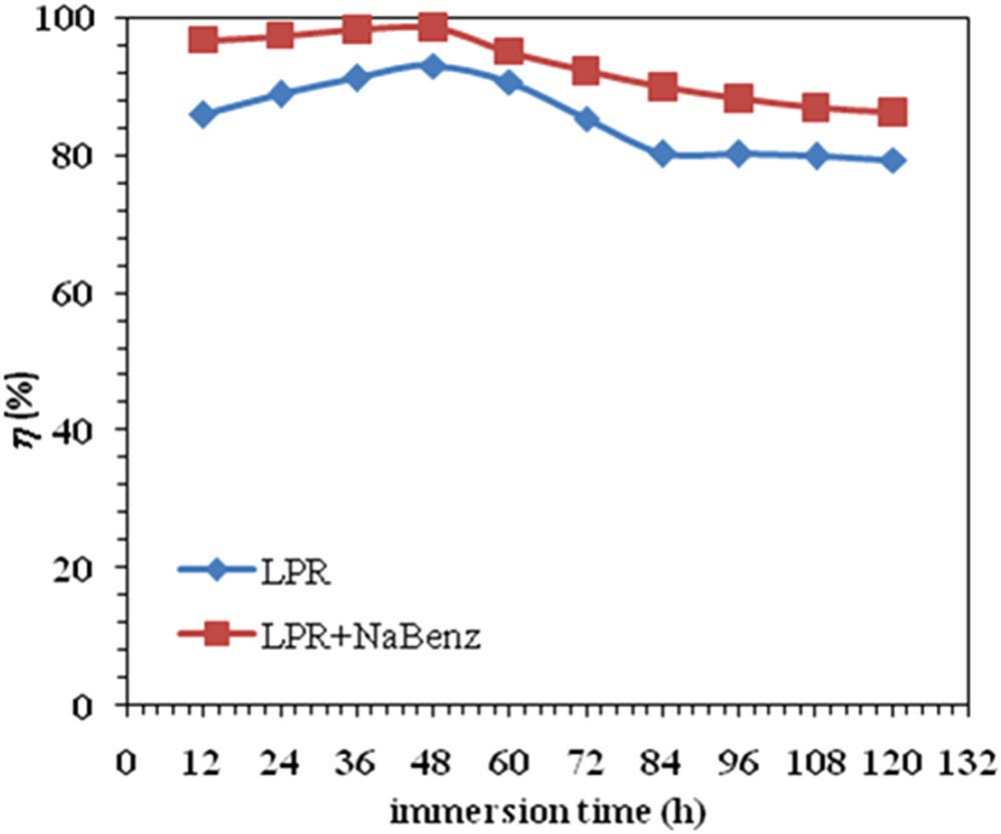
Variation of inhibition efficiency (*η*_w_%) with immersion time (*t*) for LPr and LPr + 10 ppm NaBenz.

In order to get more information about the stability of the tested inhibitor and, therefore, its lasting effect on corrosion inhibition, weight loss measurements were conducted for an extended period of 120 *h* in presence of optimum concentration of LPr and LPr + NaBenz at 30 °C. The results are presented in Fig. 8. Variation of *η*_w_ (%) with time for both systems followed the identical trend. For LPr *η*_w_ (%) increased from 82.5 to 96.82%, whereas for LPr + NaBenz mixture *η*_w_(%) was observed to increase from 96.9 to 98.51% for period extending 48 *h*. This reflects the strengthening of absorptive film on the MS surface with increasing immersion time. After time period extending 48 h a nominal decrease in inhibitors efficiency was visualized owing to the slow desorption of inhibitor molecules but LPr and LPr + NaBenz were above 80% efficient till 120 h. This confirmed the stability of LPr and LPr + NaBenz in 1M HCl over lengthened immersion period.

#### Adsorption isotherms

**Figure 9:**
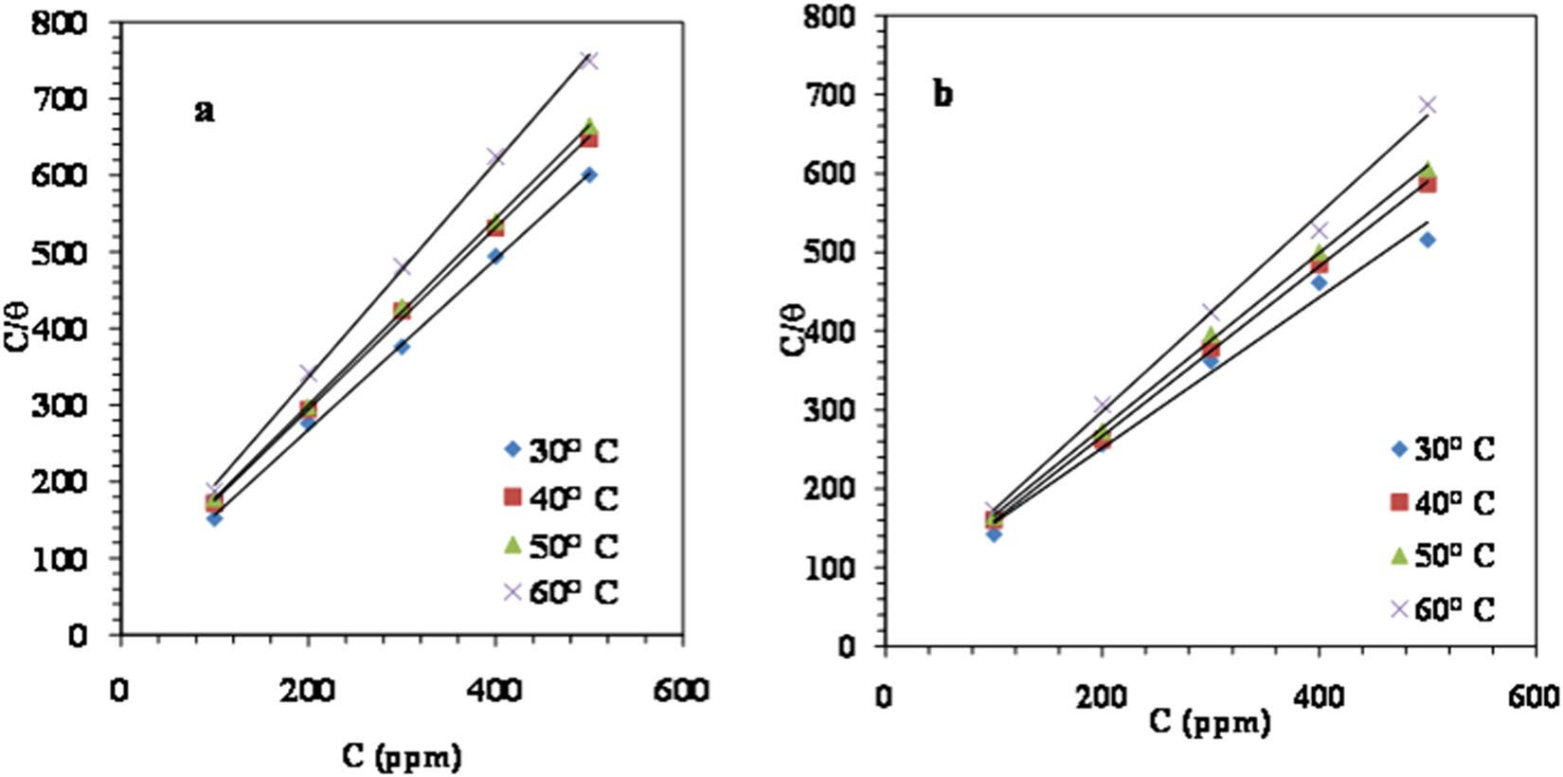
Langmuir adsorption isotherm plots for MS in 1M HCl solution containing various concentrations of (**a**) LPr (**b**) LPr + 10 ppm NaBenz at 30–60 °C.

The mechanism of inhibition of MS corrosion in 1M HCl by organic compounds can be associated with the adsorption phenomenon^45^ that would obey adsorption isotherms of different types, for example, Langmuir, Freundlich, Temkin and Frumkin^46^.The values of *θ* obtained from weight loss experiments were tested graphically to allow the fitting of a suitable isotherm and the best fit is obtained with Langmuir adsorption isotherm (Fig. 9), which is expressed by the following equation:

10 $$\frac{{\rm{C}}}{{\rm{\theta }}}=\frac{1}{{{K}}_{ads}}+{C}$$

where, *C* is inhibitor concentration and *K*_ads_ is the adsorption equilibrium constant of adsorption-desorption process. This isotherm postulates monolayer adsorption of LPr molecules on the metal surface. Regression coefficient (*R*^2^ = 0.999) (Table 6) justifies the applicability of the Langmuir isotherm model at all the studied temperature. A little deviation of slope from unity indicates the interaction between the adsorbate species on the MS surface^47^.

**Table 6:** Thermodynamic parameters of adsorption for MS in 1M HCl at 30–60 °C obtained from Langmuir adsorption isotherm.

*T* (°C)	*R* ^2^	Slope	***K***_ads_	Δ*G*°_ads_(kJ mol^−1^)
LPr				
30	0.999	1.11	0.022	−35.32
40	0.999	1.19	0.017	−35.89
50	0.999	1.22	0.018	−37.03
60	0.998	1.41	0.018	−38.27
LPr + 10 ppm NaBenz				
30	0.986	0.95	0.016	−34.53
40	0.999	1.07	0.020	−36.11
50	0.999	1.11	0.018	−37.09
60	0.995	1.25	0.020	−38.63

The value of *K*_ads_ was determined from the reciprocal of intercept of the straight line, Fig. 9. Indeed, standard Gibbs free energy Δ*G*°_ads_ of the adsorption process were obtained from following equation (11):

11 $${\rm{\Delta }}{G}_{ads}^{0}=-\,RT\,\mathrm{ln}({\rm{1}}\times {{\rm{10}}}^{{\rm{6}}}\,{{K}}_{{\rm{ads}}})$$

where 1 × 10^6^ is the concentration of water molecules expressed in ppm, *R* is the gas constant (8.314 J K^−1^ mol^−1^) and *T* is the absolute temperature (*K*)^48^.Calculated values of *K*_ads_ and Δ*G*^0^_ads_ are noted in Table 6. Values of Δ*G*°_ads_ ≤ 20 kJ/mol, in general, are in agreement with physical adsorption while values more negative than ≥40 kJ/mol signify chemical adsorption^49^.The range of Δ*G*0_ads_ values (Table 6) observed in the present work suggest that adsorption of LPr or LPr + NaBenz is not merely physical or chemical adsorption but includes a comprehensive adsorption i.e., involving both physical and chemical adsorption.

#### Activation parameters

In order to look at the effect of temperature on the corrosion reaction of MS in the presence of the LPr or LPr + NaBenz as inhibitor, Arrhenius equation was used^19,49^.

12 $$\mathrm{log}\,\nu =\,\mathrm{log}\,A-\frac{{E}_{a}}{2.303\,RT}$$

where *ν*- corrosion rate; *A*- the Arrhenius pre-exponential constant; *E*_a_- the apparent activation corrosion energy; *R*- the molar gas constant and *T*- the absolute temperature.

**Figure 10:**
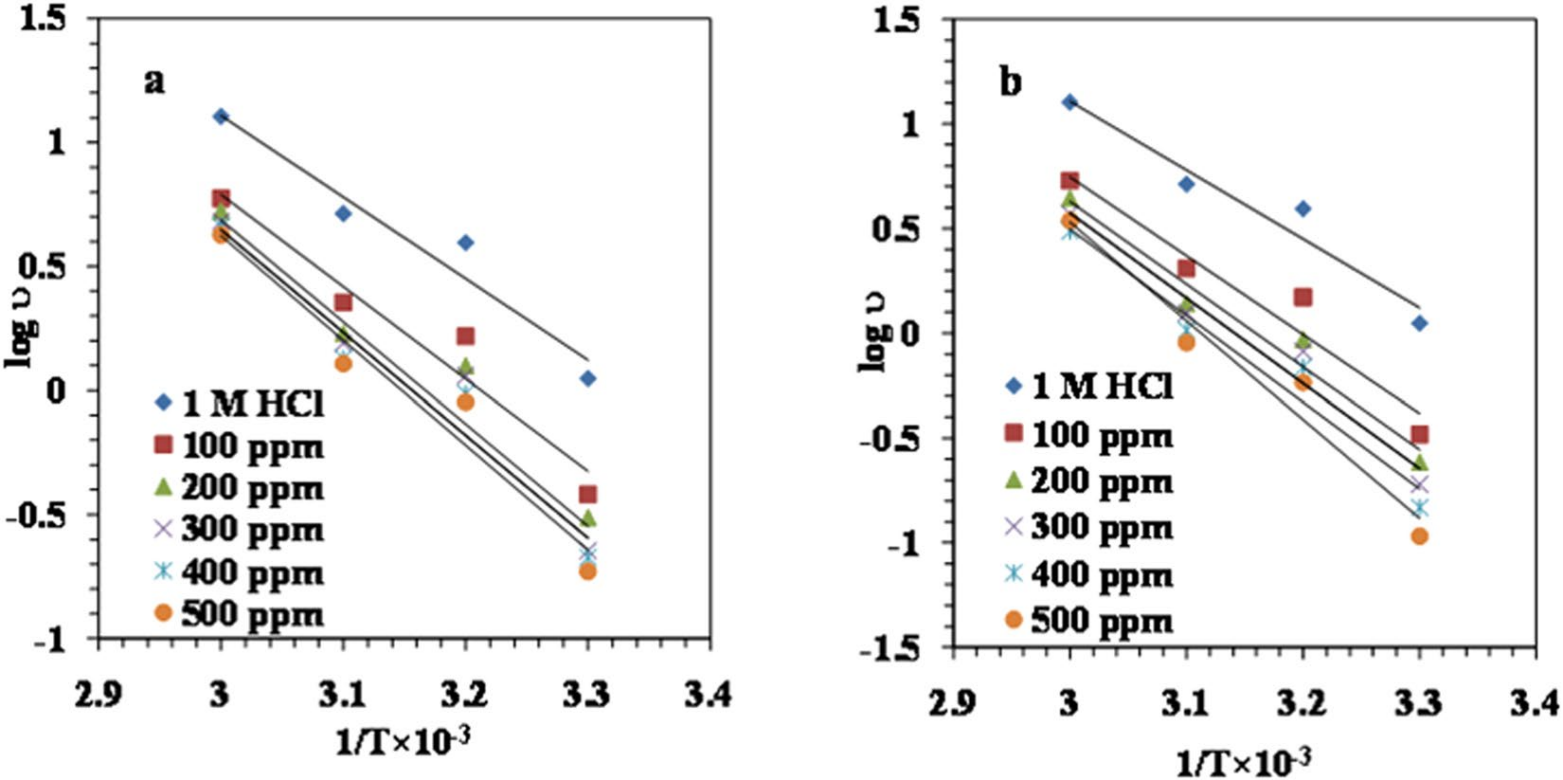
Arrhenius plots for MS in 1M HCl in the absence and presence of different concentrations of (**a**) LPr and (**b**) LPr + 10 ppm NaBenz.

The Arrhenius plots of log *ν* against 1/*T* for MS corrosion in 1M HCl solution (without and with different concentrations of LPr or LPr + NaBenz) are shown in Fig. 10a,b. The slopes of the lines were determined and the respective values of *E*_a_ were calculated from the slopes (*E*_a_ = −slope × 2.303 R). Table 7 list the calculated values of *E*_a_ for the inhibited corrosion reaction of MS. Values of *E*_a_ ranged from 71.99 to 80.81 KJ/mol and 72.11 to 118.94 KJ/mol for LPr and LPr + NaBenz, respectively. These values are higher than the value of 62.95 KJ/mol obtained for the 1M HCl solution indicating that the corrosion reaction of MS is retarded by the LPr or LPr + NaBenz and support the phenomenon of physical adsorption^50^.

**Table 7:** Activation parameters for MS corrosion in 1M HCl solution containing various concentrations of LPr and LPr + 10 ppm NaBenz.

C (ppm)	*E*_a_(kJ/mol)	ΔH*(kJ/mol)	ΔS*(kJ/mol/K)
1M HCl	62.95	60.33	0.014
Proline			
100	71.99	68.51	0.032
200	74.09	70.73	0.037
300	78.77	76.14	0.053
400	79.25	76.62	0.054
500	80.81	78.17	0.058
Proline + 10 ppm NaBenz			
100	72.11	69.50	0.034
200	75.63	73.03	0.042
300	77.77	75.15	0.048
400	79.15	76.53	0.051
500	118.94	116.32	0.172

Standard enthalpy of activation (ΔH*) and standard entropy of activation (ΔS*) were calculated by using the alternative form of Arrhenius equation:

13 $$\nu =\frac{{RT}}{{Nh}}\exp (\frac{{\rm{\Delta }}{{S}}^{\ast }}{{R}})\exp (-\frac{{\rm{\Delta }}{{H}}^{\ast }}{{RT}})$$

where *ν*- corrosion rate; *N*-the Avogadro number; *h*-the Planck’s constant; *R*-the universal gas constant and *T*-the absolute temperature.

**Figure 11:**
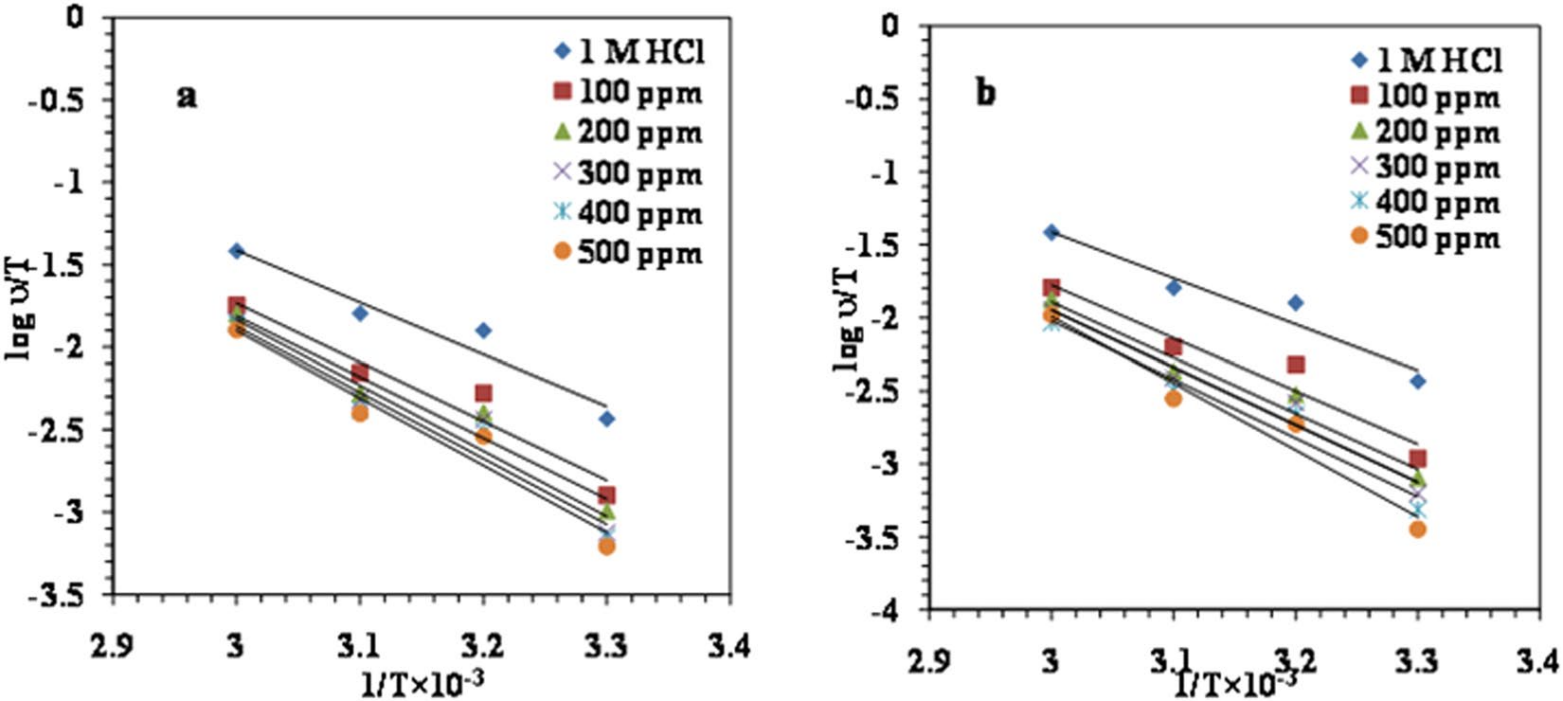
Alternative Arrhenius plots for MS in 1M HCl in the absence and presence of different concentrations of (**a**) LPr and (**b**) LPr + 10 ppm NaBenz.

The plot of log (*ν*/*T*) versus 1/*T* is given in Fig. 11. Straight line with a slope of −(ΔH*/2.303 *R*) and an intercept of {log(*R*/*Nh*) + (ΔS*/2.303 *R*)} were obtained which is used to calculate the ΔH* and ΔS* values (Table 7). Values of the ΔH* bearing positive sign indicate endothermic process and slow dissolution of MS in the presence of LPr or LPr + NaBenz^18^.The values of ΔS* are positive and slightly increased with increasing inhibitor concentration suggesting that adsorption process is accompanied by an increase in entropy. This is attributed to the adsorption of LPr or LPr + NaBenz molecules and subsequent desorption of water molecules already adsorbed on the MS surface.

14 $${{\rm{Inh}}}_{(\mathrm{sol})}+{{\rm{xH}}}_{2}{{\rm{O}}}_{({\rm{ads}})}-{{\rm{Inh}}}_{({\rm{ads}})}+{{\rm{xH}}}_{2}{{\rm{O}}}_{({\rm{sol}})}$$

where x represents the number of water molecules replaced by one molecule of adsorbed inhibitor molecule. As the values of ΔS* represent the algebraic sum of adsorption of inhibitor (resulting in lowering of solute entropy) and desorption of water molecules(resulting in increase of solvent entropy), the positive values of the entropy data imply that more water molecules are leaving the MS surface with the subsequent replacement with inhibitor molecules^51^.

### Spectroscopic studies

#### UV–vis spectroscopic analysis

**Figure 12:**
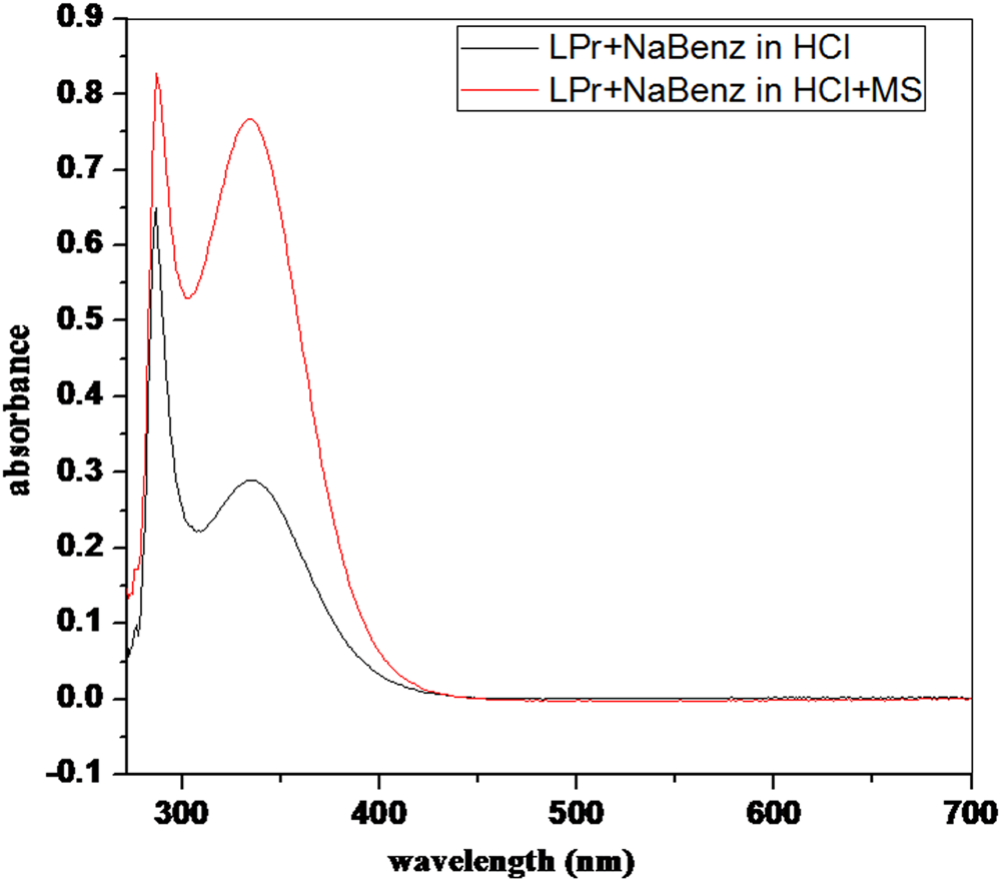
UV-vis spectra of acidic solutions of 500 ppm LPr + 10 ppm NaBenz before and after 6 h immersion of mild steel.

After completion of weight loss experiment in presence of optimum concentration of LPr + NaBenz, the thin film formed on the MS surface was scraped, collected, dissolved in methanol and subjected to UV-visible study. Figure 12 shows the UV−visible spectrum of LPr + NaBenz and scraped sample. A visible difference in the two spectrums exists suggesting the existence of [organic inhibitor-Fe] on the MS in acid solution. Spectrum of LPr + NaBenz solution, prior to immersion of MS, showed absorption peaks at 286 and 334 nm with corresponding absorbance value of 0.64 and 0.28, respectively. The spectrum of scraped sample shows a slight blue shift in the maximum absorption wavelength to 288 and 335 nm with corresponding higher absorbance of 0.82 and 0.76. This indicates the presence of an interaction of π-electrons with vacant d-orbitals of the metal and/or an interaction between unshared electron pairs in the inhibitor molecule and vacant d-orbitals of the metal.

#### FT-IR spectroscopy

**Figure 13:**
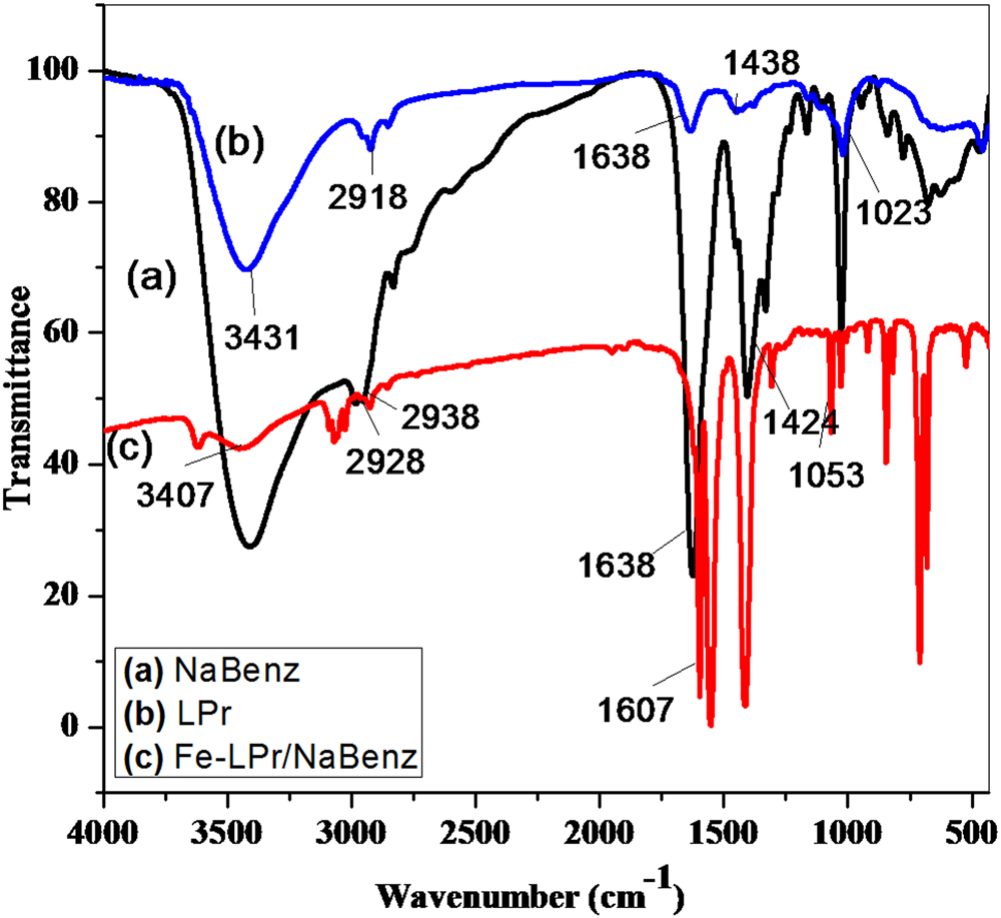
FT-IR spectra of pure LPr, NaBenz and LPr + NaBenz adsorbed on mild steel surface after 6 h immersion.

The FT-IR characteristics of the LPr and NaBenz and the LPr + NaBenz film formed on the MS surface are presented in Fig. 13a–c. FT-IR spectra of LPr showed characteristic peaks for N–H and C=O stretching vibrations around 3431 and 1638 cm^−1^, respectively. The peaks of 2918 and 1424 cm^−1^, respectively correspond to the stretching vibration of -CH_2_- and bending vibration of C-H. The stretching vibration of C=O results in the peak at 1638 cm^−1^ and the peak of 1023 cm^−1^ and 1053 (for LPr and NaBenz, respectively) is ascribed to the C-O stretching vibration. Furthermore, Fig. 13(c) shows the similar FT-IR spectrum, and the peak regions of the adsorptive film are basically consistent with that of pure LPr and pure NaBenz. However, the intensities of the peak at 3431, 1638 cm^−1^ become weaker (3407 and 1607 cm^−1^) after the adsorption of LPr + NaBenz mixture. This phenomenon may be the result of the interaction of some active components of LPr + NaBenz and the mild steel, forming a layer of film on the MS surface^52^.

#### Surface morphological studies

**Figure 14:**
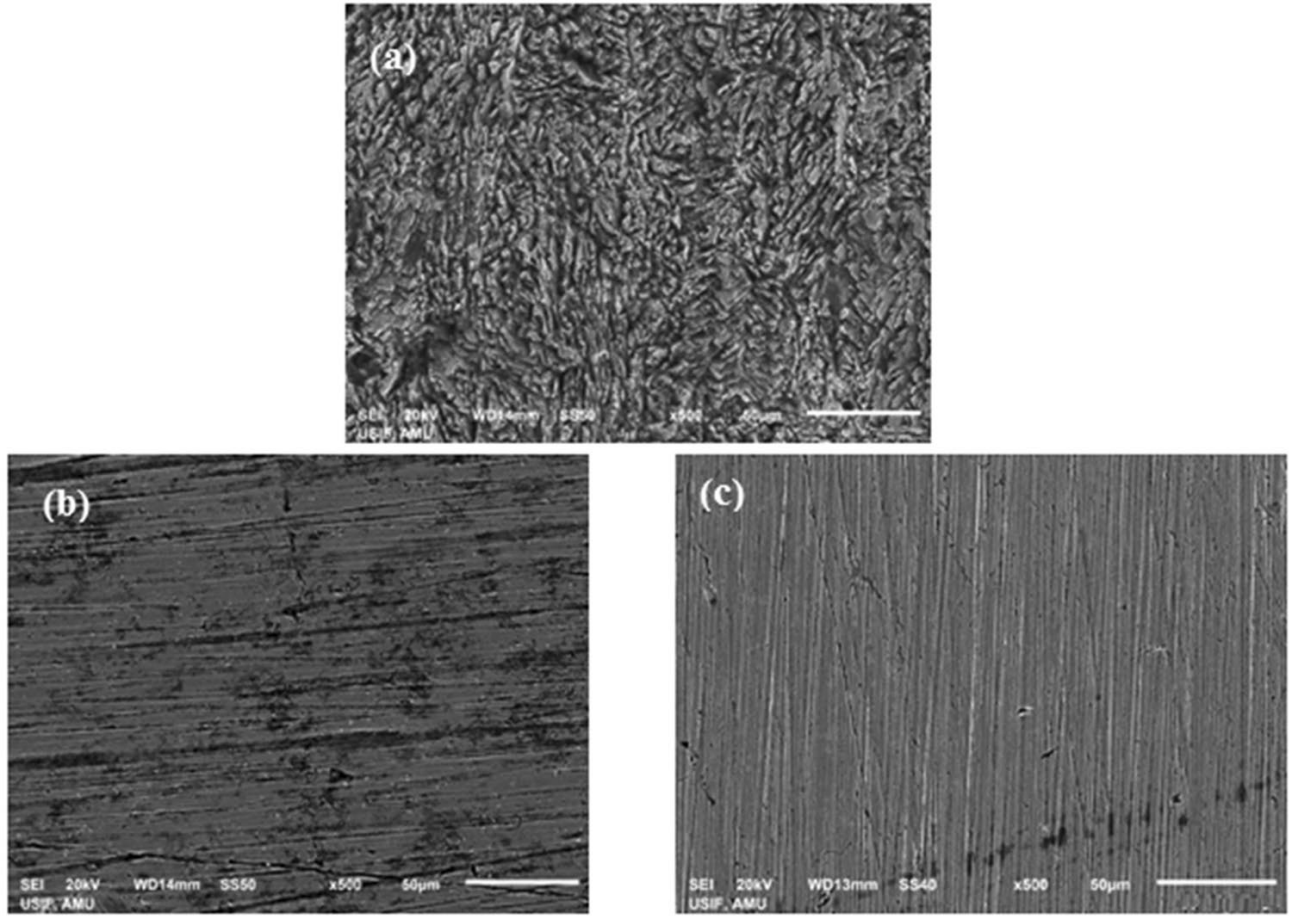
SEM images of the MS after 6 h immersion in 1M HCl solution: (**a**) in 1M HCl, (**b**) in 1M HCl solution with LPr (**c**) in 1M HCl solution with LPr + 10 ppm NaBenz.

Surface morphology of MS specimens prior to and after immersion in uninhibited and inhibited HCl solution were evaluated using SEM and the resultant photomicrographs are shown in Fig. 14a–c. In HCl solution with no inhibitor, the specimen surface is highly damaged due to the attack of corrosive acid solution (Fig. 14a). In presence of LPr the surface heterogeneity is markedly suppressed due to the formation of a protective covering by the adsorbed LPr molecules (Fig. 14b). The addition of NaBenz to LPr inhibited acid solution caused additional surface coverage, which lead to formation of a more complete film and as a result further improvement in the surface smoothness, was observed (Fig. 14c). The morphology resembled to that of a freshly polished MS surface.

#### Density functional theory

**Figure 15:**
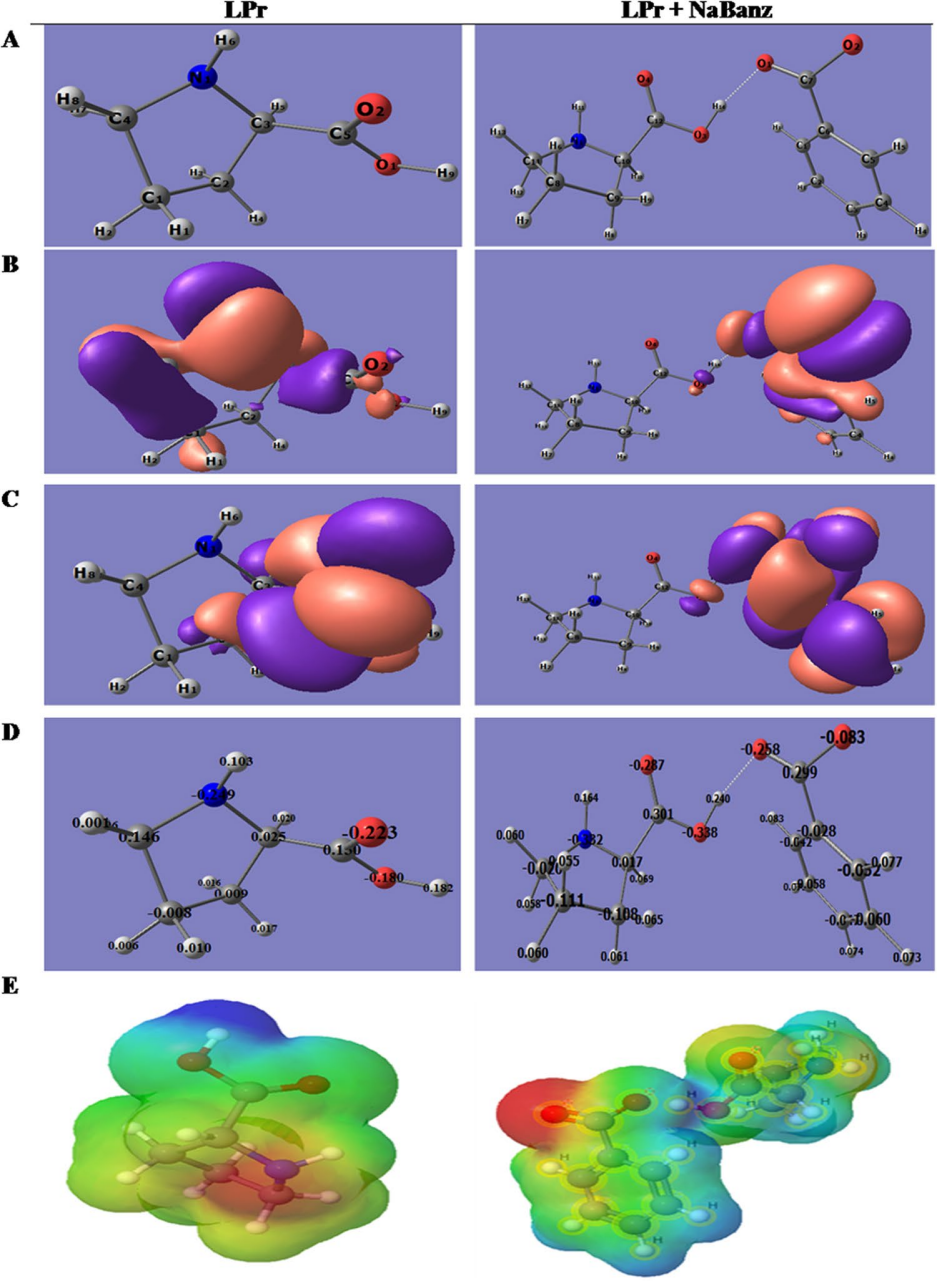
Quantum chemical results of Proline in the absence and prsence of NaBenz calculated by the ORCA programme: (**A**) optimized molecular structure, (**B**) HOMO; (**C**) LUMO (**D**) Mulliken charges (**E**) MEP.

DFT have been identified as worthwhile tool for elucidating the molecular structure and relative reactivity of the molecule. The LPr molecules alone and in combination with NaBenz were optimized to ground state with minimum energy and the optimized geometry, frontier molecular orbital’s density distribution (HOMO and LUMO), mulliken’s charge density and the MEP for LPr and LPr + NaBenz are shown in Fig. 15A–E and corresponding computed parameters are depicted in Table 8. It is apparent from the Fig. 15B that the electron distribution of HOMO orbitals of LPr molecule are mainly localized on the N and C atoms and LPr + NaBenz are localized principally on the -COO^−^ group. These sights might favor electron donations to vacant d-orbitals of Fe (MS) during donor-acceptor interactions. The LUMO energy orbitals are localized on the -COOH of LPr while in LPr + NaBenz, LUMO electron density spreads on the phenyl ring in the molecule (Fig. 15C).

**Table 8:** The calculated quantum chemical parameters for studied inhibitors obtained using DFT at the B3LYP def2-SVP basis set.

Inhibitor	***E***_HOMO_	***E***_LUMO_	Δ***E***	***E***_t_	***I***	***A***	***χ***	***η***	Δ***N***
LPr	3.695	0.075	−3.62	−10901.82	−3.695	−0.075	−1.81	−1.885	2.558
LPr + NaBenz	5.918	−2.083	−8.001	−18061.09	−5.918	2.083	−4.001	−1.918	2.541

Based on the frontier molecular orbital theory, *E*_HOMO_ is typically correlated with the electron donating capability of the molecule and offers considerable insight about the sites or segments of the evaluated molecules which can serve as electron donor during the adsorption on the metal surface. The order of *E*_HOMO_ is LPr < Lpr + NaBenz. *E*_LUMO_ is associated with the electron accepting capability of the molecule and lower the value better is the potential of the molecule to receive electrons from the metal surface and here values follow the order: LPr > Lpr + NaBenz. The separation energy (Δ*E*_LUMO-HOMO_) is a measure of reactivity of a molecule. Thus, a molecule with minimal separation energy are associated with high chemical reactivity and high inhibition efficiency, as the excitation energy to remove an electron from the last occupied orbital will be low. A smaller HOMO–LUMO gap signifies high stability for the compound in the complex formed with the MS surface. Δ*E*_LUMO-HOMO_ value for LPr + NaBenz is lower as compared to LPr alone. The molecules with the high inhibition of corrosion have lower *E*_t_ values and from the Table 6, it is clear that the *E*_t_ value for LPr + NaBenz is less than the *E*_t_ value for LPr alone. According to Koopmen’s theorem, the values of *E*_HOMO_ and *E*_LUMO_ of the inhibitor molecule are association with the ionization potential (*I*) and the electron affinity (*A*), respectively. The values of *I* and *A* are defined as −*E*_HOMO_ and −*E*_LUMO_, respectively and the obtained values are used to calculate the global hardness (*η*) and the electronegativity (*χ*).

15 $$\eta =\frac{I-A}{2}$$

16 $$\chi =\frac{I+A}{2}$$

Calculated values of *χ* are also mentioned in Table 6, which denotes the tendency of an atom to attract the shared pair of electron towards itself. The value of *χ* for LPr + NaBenz is lower than the value for LPr alone. Absolute hardness, *η* determines both the stability and reactivity of a molecule which suggests the resistivity of the inhibitor for the physical adsorption process. Soft molecules with small energy gaps are far more reactive than hard ones with large energy gaps, as they could readily offer electrons to an acceptor.

The fraction of electrons transferred (Δ*N*) can be expressed as follows.

17 $${\rm{\Delta }}N=\frac{{\chi }_{Fe}+{\chi }_{inh}}{2({\eta }_{Fe}+{\eta }_{inh})}$$

where *χ*_Fe_ and *χ*_*i*nh_ represent the electronegativity and the *η*_Fe_ and *η*_inh_ represent the absolute hardness of iron and the inhibitor molecules, respectively. The theoretically calculated value of *χ*_Fe_ for iron metal is 7 eV mol^−1^ and the *η*_Fe_ is 0 eV mol^−1^. These values are appropriately substituted to calculate Δ*N*. Values of Δ*N* exhibit the path of the electron transfer between inhibitor and metal surface. The Δ*N* exhibit the inhibitive performance of the inhibitors resulted from electron donations. In the current investigation, Δ*N* values are greater than zero indicating electron transfer from the inhibitor to the MS surface^53^.

Mulliken’s charge on the atoms provides the insight on the selectivity of molecule interacting with metal surface. In general, the atoms which carries negative charges have inherent tendency to donate its electrons to the metal surface^54^. Figure 15D shows the Mulliken’s charge density on the molecules. For the LPr molecule alone, it is observed that the hetero atom N, O along with C_1_ atoms carry negative charges, which confirms that LPr can give lone pair of electron form these donor sites to the metal’s vacant d-orbital. Furthermore, it is also observed that most of the C atoms are carrying positive charges, these are the sites at which LPr molecule accept electrons from metal surface. In case of LPr + NaBenz heteroatoms N, O anlong with some C carry negative charge while other C carry positive charge.

MEPs mapping were also obtained for the investigated molecule to define the sites of electrophilic attack in the adsorption process with metal surface which have been defined by the color coding; the red and blue color shows respectively the electron rich and deficient regions while green colour represents the neutral part of the molecule. Figure 15E, shows the negative charge density localized on the sites involving high ability in metal coordination.

## Conclusion

1. LPr mixed with NaBenz acts as highly efficient sustainable corrosion inhibiting formulation for MS in 1M HCl solution.
2. Inhibition efficacy of LPr is synergistically improved in presence of NaBenz (10 ppm) to the aggressive acid solution.
3. Evaluation of polarization parameters suggested that both LPr and LPr mixed with NaBenz act as mixed type inhibitor with more control on cathodic reaction.
4. Evaluation of impedance parameters indicated the formation of protective layer at the MS/corrodent interface.
5. Spectroscopic studies like FT-IR and the UV-vis showed interaction of the inhibitors with the MS surface suggesting a possible inhibitor/MS complex formation.
6. SEM micrographs revealed that surface heterogeneity of MS was considerably reduced in presence of LPr and LPr mixed with NaBenz giving clear indication of their adsorption on steel surface and adequate protection in 1M HCl solution.
7. Thermodynamic and kinetic parameters suggested that the process of adsorption of LPr or LPr + NaBenz mixture is spontaneous, comprehensive, and endothermic and caused an increase in the entropy of the system.
8. Quantum chemical calculations were performed on LPr and LPr mixed with NaBenz and different molecular structural parameters were computed and discussed.
